# Supplementary material for: Reading Reshapes Stimulus Selectivity in the Visual Word Form Area
Source: eNeuro. 2024 Jul 25;11(7):ENEURO.0228-24.2024. doi: 10.1523/ENEURO.0228-24.2024 (PMC11285298; doi:10.1523/ENEURO.0228-24.2024)
Supplement: Figure 1-1 — All words and pseudowords used in the experiment. Download Figure 1-1, DOCX file. [file eneuro-11-ENEURO.0228-24.2024-s001.docx]

| **Real Words** | | | | | **Pseudowords** | | | | |
| --- | --- | --- | --- | --- | --- | --- | --- | --- | --- |
| fall | fair | heat | talk | bulb | onll | kide | tiet | cais | spon |
| dung | feet | game | stop | jeep | soad | evey | iraw | onod | mext |
| warm | rate | wide | send | role | sood | veen | geal | alst | nean |
| team | cold | pads | wait | maze | pust | reen | gope | woud | sain |
| wall | bats | nets | bald | owed | cich | sont | rown | fole | nage |
| dual | leak | hush | feat | fake | edea | hing | rops | gomy | fize |
| cart | lose | word | baby | fish | hane | yeal | onte | mowl | firl |
| muck | news | lily | ness | hell | tris | sloy | wist | mank | alat |
| flap | paid | cans | flee | fast | bour | tand | yext | wiky | diew |
| foam | pens | moss | lead | none | surn | nost | whis | lato | weat |
| ants | near | nude | kept | bows | nery | samb | gove | fook | dasy |
| size | bees | crap | lack | safe | coar | doel | tuch | hece | jure |
| oath | sort | cops | butt | lost | halp | sind | woth | lood | inlk |
| line | main | ache | menu | show | eved | moad | aung | higs | fike |
| bunk | lone | pans | view | free | moen | pich | houd | alar | juch |
| flat | peer | mint | grow | dark | welp | ment | wele | pase | sani |
| poor | club | chub | axis | drum | youd | jook | frew | ovew | dake |
| lung | wind | cord | boot | note | toat | lely | hais | inen | purd |
| hind | lads | seat | melt | lady | vero | mang | derm | leat | lind |
| edge | care | step | wife | norm | cass | yeen | neny | vass | aror |
| move | lied | deed | coin | lent | sork | toul | dook | juru | tind |
| hail | pier | cosy | aids | oats | mear | dowl | mard | wien | grur |
| mass | bust | male | fuse | lush | lery | pign | mune | foor | gool |
| oral | liar | area | lain | dame | hery | basy | wesk | sond | egen |
| plan | race | gaps | jars | walk | ween | clus | cerm | knto | wany |
| jets | type | gone | stay | arid | bory | blso | palk | rean | tery |
| junk | lids | hair | hour | oils | rith | golt | goor | werr | parm |
| fire | thin | fare | ways | sent | frme | tast | hony | mave | elsk |
| lord | comb | huge | shot | milk | heah | lesk | milm | wene | blus |
| gets | hope | tree | bred | cult | feld | iney | huss | oner | fing |
| bass | loss | jerk | seem | call | lons | liet | traw | cont | dopy |
| test | buys | rock | east | dash | fack | soll | havy | lome | arom |
| ones | isle | wild | mine | desk | unth | yoom | muro | kner | lish |
| coup | hymn | dear | ally | rise | eang | como | helf | roso | masy |
| dose | horn | pass | grip | duly | woen | sath | sest | shem | wead |
| jazz | caps | aide | west | ball | cand | thil | timp | lelf | eart |
| born | neck | fine | sign | thus | thow | eath | mish | bere | foon |
| hall | dead | foot | loaf | bolt | loat | rell | sids | dath | boin |
| bump | town | died | haul | late | rata | tood | terl | dort | foss |
| boys | gale | live | loop | dump | lous | clat | darl | tere | pook |
| dole | drab | limb | heir | chew | fift | vech | tirl | loof | werk |
| army | bake | skin | gasp | fade | nore | riss | toto | frow | loor |
| knot | deaf | fads | haze | bank | bood | beey | gaid | lact | fith |
| nuns | gold | bony | rest | pain | seef | frop | bext | shus | gide |
| huts | cane | doom | duck | meat | thad | hent | onch | hasy | thip |

**Extended Data Figure 1-1:** All words and pseudowords used in the experiment.
