# Supplementary figures and images for: Reading Reshapes Stimulus Selectivity in the Visual Word Form Area
Source: eNeuro. 2024 Jul 25;11(7):ENEURO.0228-24.2024. doi: 10.1523/ENEURO.0228-24.2024 (PMC11285298; doi:10.1523/ENEURO.0228-24.2024)

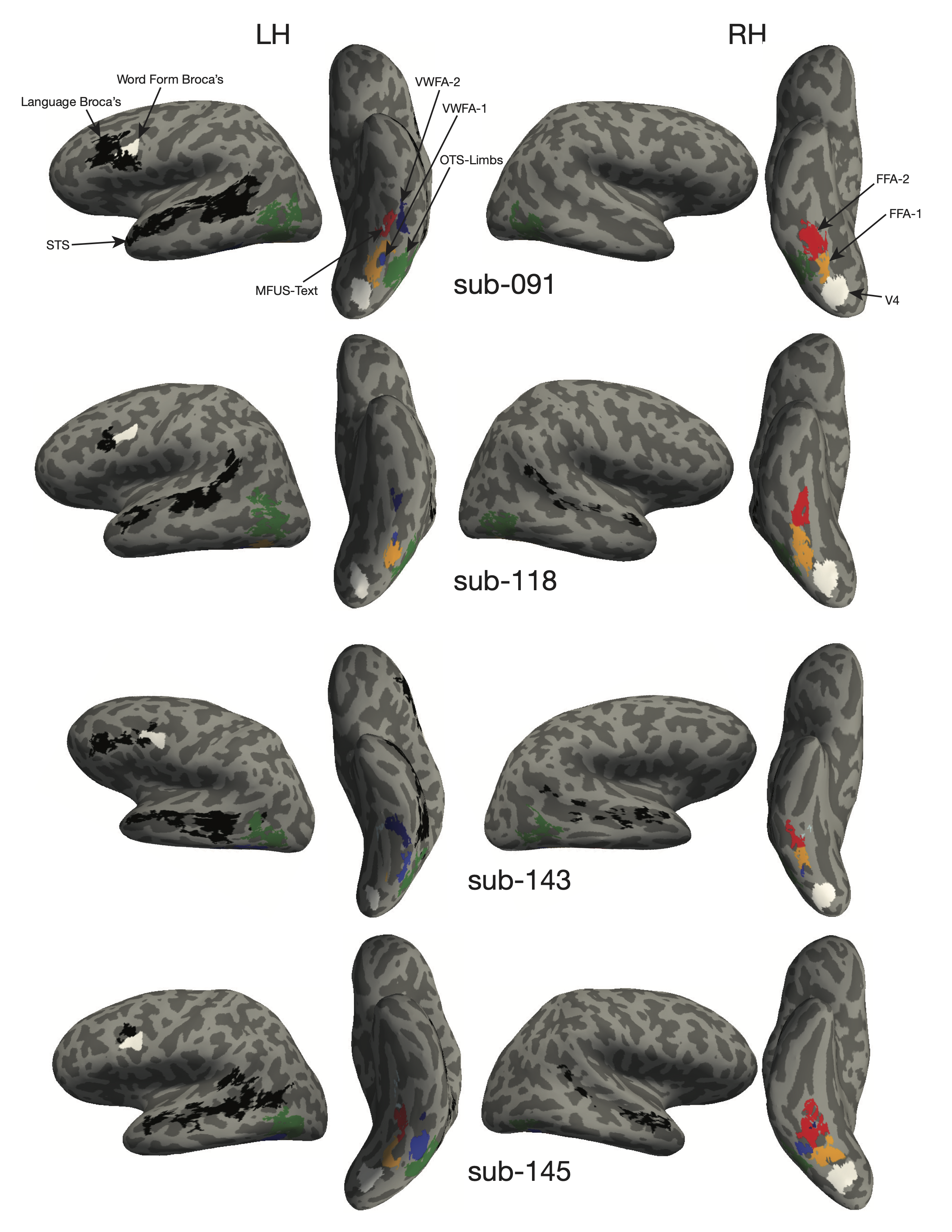

Supplement: Figure 1-2 — Regions of interest in individual subject anatomical surface space. Download Figure 1-2, TIF file. [file eneuro-11-ENEURO.0228-24.2024-s002.tif]

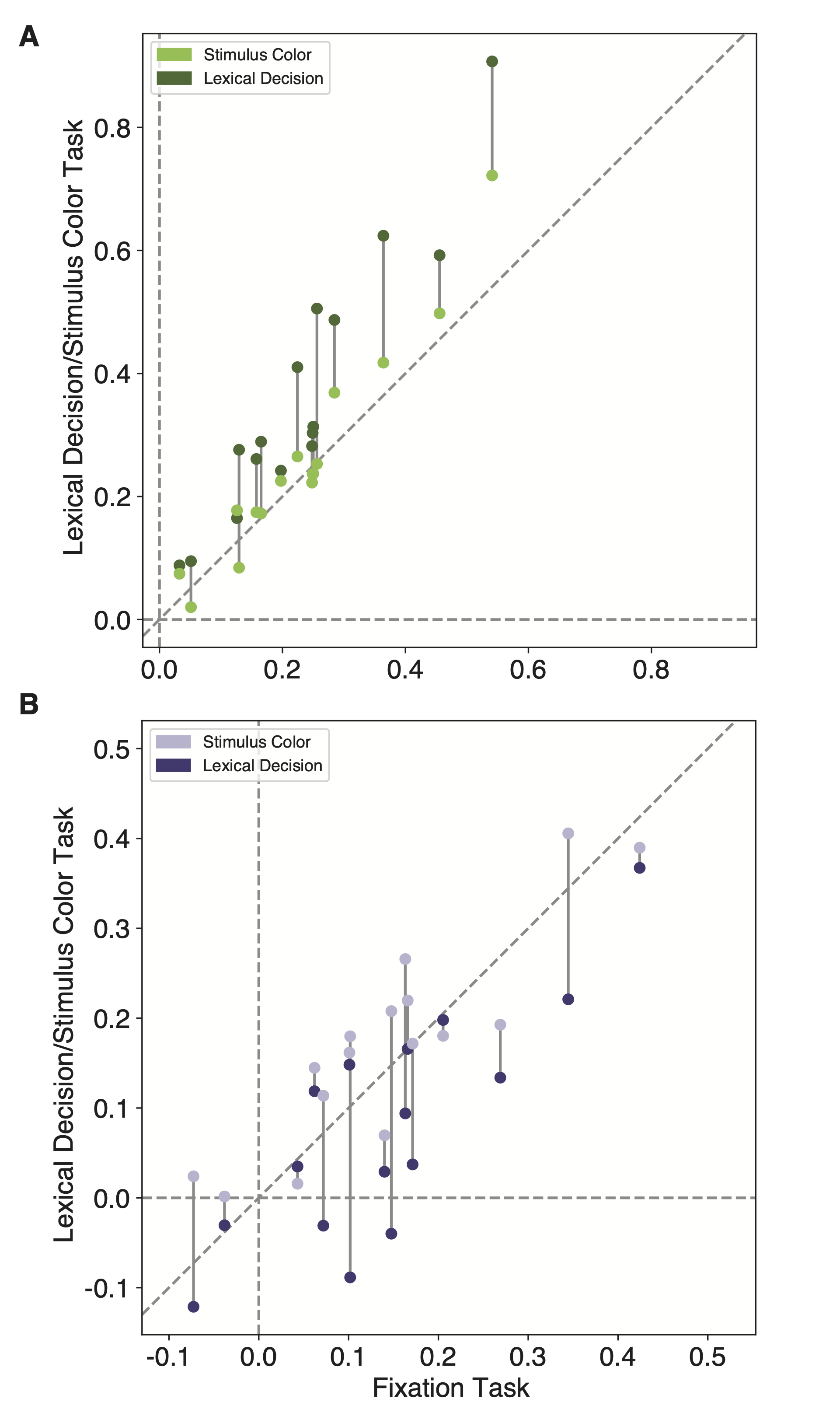

Supplement: Figure 2-1 — Individual participants’ mean beta responses in the left VWFA (VWFA-1 and VWFA-2 together) for A) text (real words and pseudowords) and B) false fonts. In each panel, each participant contributes two points that are connected by a vertical line. The horizontal position of each point represents that subject’s VWFA response during the fixation task. The vertical position of the darker points represents the response during the lexical decision task, and the vertical position of the lighter points represents the stimulus color task. Download Figure 2-1, TIF file. [file eneuro-11-ENEURO.0228-24.2024-s004.tif]

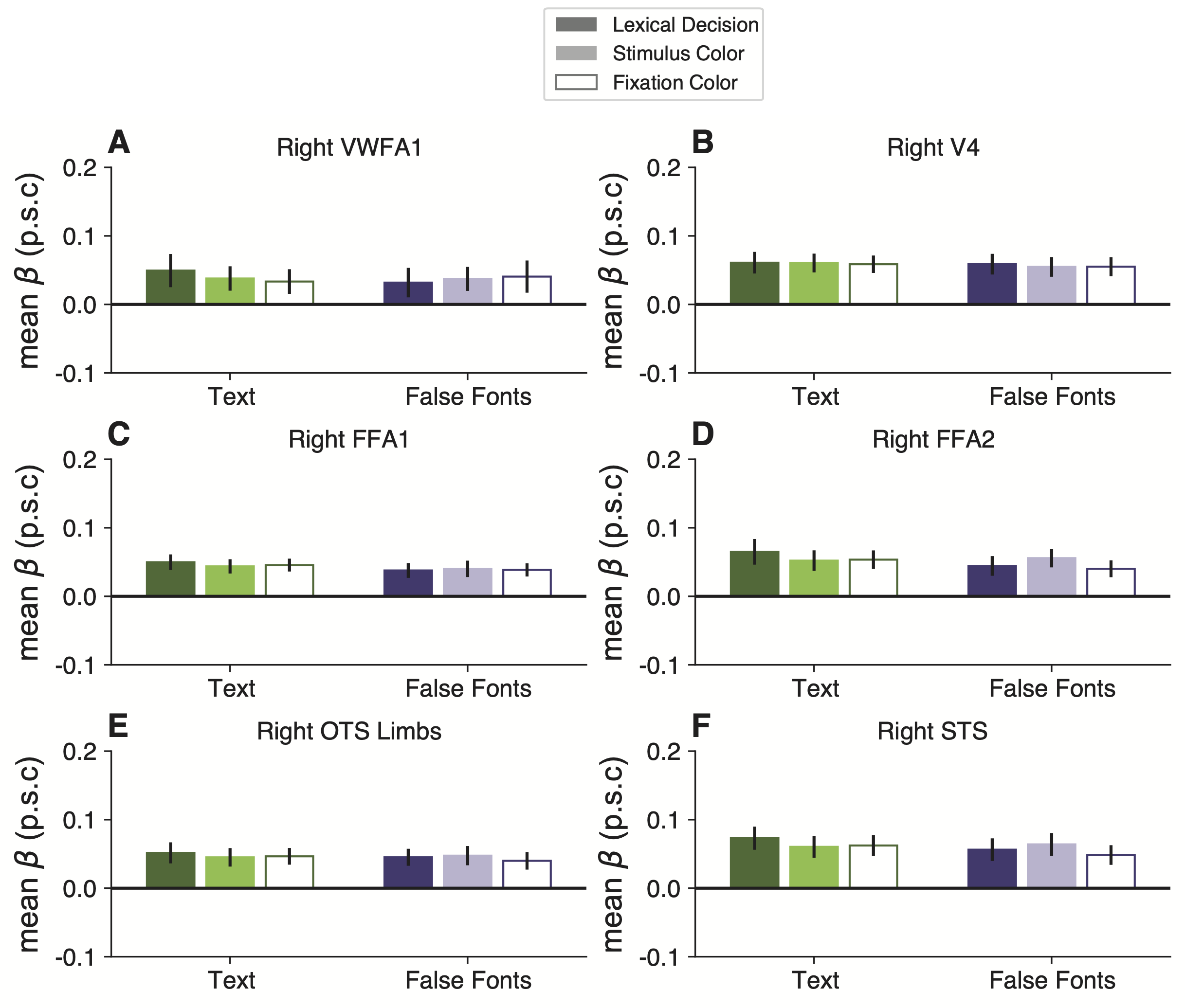

Supplement: Figure 3-2 — Mean BOLD percent signal change as a function of stimulus and task types in control ROIs in the right hemisphere. Error bars represent standard error of the mean. Download Figure 3-2, TIF file. [file eneuro-11-ENEURO.0228-24.2024-s006.tif]
