# Supplementary material for: Reading Reshapes Stimulus Selectivity in the Visual Word Form Area
Source: eNeuro. 2024 Jul 25;11(7):ENEURO.0228-24.2024. doi: 10.1523/ENEURO.0228-24.2024 (PMC11285298; doi:10.1523/ENEURO.0228-24.2024)
Supplement: Table 2-1 — Statistics for stimulus and task differences for VWFA-1 and -2, reported separately. Download Table 2-1, DOCX file. [file eneuro-11-ENEURO.0228-24.2024-s003.docx]

| **ROI** | **Stimulus** | **Task** | **Mean ΔPSC** | **SEM** | **95% CI** | **t-value** | **BF** | **p-value** |
| --- | --- | --- | --- | --- | --- | --- | --- | --- |
| VWFA1 | Text | LD - FC | 0.15 | 0.02 | 0.09, 0.2 | 6.05 | 44.95 | <0.001 |
|  |  | LD - SC | 0.12 | 0.02 | 0.07, 0.17 | 5.77 | 34.29 | <0.001 |
|  |  | SC - FC | 0.03 | 0.02 | 0.02, 0.08 | 1.47 | 1 | 0.147 |
|  | False Fonts | LD - FC | -0.06 | 0.03 | -0.14, 0.01 | 2.03 | 2 | 0.07 |
|  |  | LD - SC | -0.09 | 0.03 | -0.16, -0.02 | 3.31 | 6.35 | 0.004 |
|  |  | SC - FC | 0.03 | 0.03 | -0.04, 0.1 | 1.04 | 1.08 | 0.3 |
| VWFA2 | Text | LD - FC | 0.12 | 0.02 | 0.07, 0.16 | 6.5 | 527.66 | <0.001 |
|  |  | LD - SC | 0.1 | 0.02 | 0.05, 0.14 | 5.67 | 1603.96 | <0.001 |
|  |  | SC - FC | 0.02 | 0.02 | 0.01, 0.07 | 1.35 | 0.84 | 0.18 |
|  | False Fonts | LD - FC | -0.07 | 0.02 | -0.12, 0.01 | 2.71 | 8.79 | 0.01 |
|  |  | LD - SC | -0.09 | 0.02 | -0.15, -0.03 | 3.88 | 32.28 | 0.001 |
|  |  | SC - FC | 0.03 | 0.02 | -0.03, 0.08 | 1.05 | 0.57 | 0.3 |

**Extended Data Table 2-1:** Statistics for stimulus and task differences for VWFA-1 and -2, reported separately.
