# Supplementary material for: Reading Reshapes Stimulus Selectivity in the Visual Word Form Area
Source: eNeuro. 2024 Jul 25;11(7):ENEURO.0228-24.2024. doi: 10.1523/ENEURO.0228-24.2024 (PMC11285298; doi:10.1523/ENEURO.0228-24.2024)
Supplement: Figure 3-1 — Statistics for task effects on responses to each stimulus type for all control ROIs in the left hemisphere. P-values are corrected for multiple comparisons using FDR correction. All p-values were corrected for post-hoc tests within ROIs, and then concatenated and corrected for multiple comparisons across ROIs. Adjusted P-values are reported in the final column of the table. Download Figure 3-1, DOCX file. [file eneuro-11-ENEURO.0228-24.2024-s005.docx]

| **ROI** | **Stimulus** | **Task** | **Mean ΔPSC** | **SEM** | **95% CI** | **t-value** | **BF** | **p-value** |
| --- | --- | --- | --- | --- | --- | --- | --- | --- |
| FFA1 | Text | LD - FC | -0.02 | 0.01 | -0.04, 0.02 | 1.15 | 0.44 | 0.26 |
|  |  | LD - SC | -0.03 | 0.01 | -0.06, 0.001 | 2.38 | 1.86 | 0.06 |
|  |  | SC - FC | 0.01 | 0.01 | -0.01, 0.04 | 1.13 | 0.86 | 0.26 |
|  | False Fonts | LD - FC | -0.005 | 0.02 | -0.05, 0.04 | 0.31 | 0.29 | 0.76 |
|  |  | LD - SC | 0.009 | 0.02 | -0.03, 0.05 | 0.54 | 0.28 | 0.76 |
|  |  | SC - FC | -0.01 | 0.02 | -0.05, 0.03 | 0.86 | 0.32 | 0.76 |
| FFA2 | Text | LD - FC | 0.025 | 0.02 | -0.01, 0.06 | 1.65 | 0.6 | 0.72 |
|  |  | LD - SC | 0.014 | 0.02 | -0.02, 0.05 | 0.95 | 0.34 | 0.72 |
|  |  | SC - FC | 0.012 | 0.02 | -0.02, 0.05 | 0.81 | 0.7 | 0.72 |
|  | False Fonts | LD - FC | -0.03 | 0.02 | -0.08, 0.02 | 1.48 | 1.42 | 0.72 |
|  |  | LD - SC | -0.04 | 0.02 | -0.09, 0.01 | 2.07 | 1.55 | 0.72 |
|  |  | SC - FC | 0.01 | 0.02 | 0.04, 0.06 | 0.55 | 0.39 | 0.86 |
| OTS-Limbs | Text | LD - FC | 0.02 | 0.01 | -0.01, 0.04 | 1.45 | 0.5 | 0.72 |
|  |  | LD - SC | 0.02 | 0.01 | -0.005, 0.043 | 1.96 | 0.67 | 0.72 |
|  |  | SC - FC | -0.003 | 0.01 | -0.03, 0.02 | 0.28 | 0.29 | 0.91 |
|  | False Fonts | LD - FC | -0.014 | 0.01 | -0.05, 0.02 | 0.95 | 0.34 | 0.72 |
|  |  | LD - SC | -0.033 | 0.01 | -0.07, -0.001 | 2.52 | 1.25 | 0.48 |
|  |  | SC - FC | 0.019 | 0.01 | -0.013, 0.05 | 1.46 | 0.62 | 0.72 |
| STS | Text | LD - FC | 0.009 | 0.007 | -0.009, 0.03 | 1.2 | 0.5 | 0.72 |
|  |  | LD - SC | 0.01 | 0.007 | -0.008, 0.03 | 1.35 | 0.7 | 0.72 |
|  |  | SC - FC | -0.001 | 0.007 | -0.02, 0.02 | 0.15 | 0.27 | 0.96 |
|  | False Fonts | LD - FC | 0.01 | 0.01 | -0.01. 0.04 | 1.11 | 0.41 | 0.4 |
|  |  | LD - SC | -0.002 | 0.01 | -0.03, 0.02 | 0.16 | 0.26 | 0.87 |
|  |  | SC - FC | 0.01 | 0.01 | -0.1, 0.04 | 1.28 | 0.67 | 0.4 |
| V4 | Text | LD - FC | -0.01 | 0.009 | -0.04, 0.01 | 1.38 | 0.52 | 0.81 |
|  |  | LD - SC | -0.009 | 0.009 | -0.03, 0.14 | 0.95 | 0.33 | 0.81 |
|  |  | SC - FC | -0.004 | 0.009 | -0.03, 0.02 | 0.42 | 0.29 | 0.89 |
|  | False Fonts | LD - FC | 0 | 0.01 | -0.03, 0.03 | 0.03 | 0.26 | 0.98 |
|  |  | LD - SC | -0.001 | 0.01 | -0.03, 0.03 | 0.05 | 0.26 | 0.98 |
|  |  | SC - FC | 0.001 | 0.01 | -0.03, 0.03 | 0.08 | 0.26 | 0.98 |
| MFUS-Text | Text | LD - FC | 0.07 | 0.02 | 0.02, 0.12 | 3.68 | 1.99 | 0.07 |
|  |  | LD - SC | 0.06 | 0.02 | 0.01, 0.11 | 3.29 | 2.14 | 0.07 |
|  |  | SC - FC | 0.01 | 0.02 | -0.04, 0.06 | 0.54 | 0.32 | 0.86 |
|  | False Fonts | LD - FC | -0.04 | 0.03 | -0.11, 0.02 | 1.6 | 0.53 | 0.72 |
|  |  | LD - SC | -0.06 | 0.03 | -0.12, 0.01 | 2.1 | 1.44 | 0.72 |
|  |  | SC - FC | 0.012 | 0.03 | -0.05, 0.08 | 0.47 | 0.28 | 0.89 |

**Extended Data Figure 3-1:** Statistics for task effects on responses to each stimulus type for all control ROIs in the left hemisphere. P-values are corrected for multiple comparisons using FDR correction. All p-values were corrected for post-hoc tests within ROIs, and then concatenated and corrected for multiple comparisons across ROIs. Adjusted P-values are reported in the final column of the table.
